# Translating statistical species-habitat models to interactive decision support tools

**DOI:** 10.1371/journal.pone.0188244

**Published:** 2017-12-13

**Authors:** Lyndsie S. Wszola, Victoria L. Simonsen, Erica F. Stuber, Caitlyn R. Gillespie, Lindsey N. Messinger, Karie L. Decker, Jeffrey J. Lusk, Christopher F. Jorgensen, Andrew A. Bishop, Joseph J. Fontaine

**Affiliations:** 1 Nebraska Cooperative Fish & Wildlife Research Unit, University of Nebraska-Lincoln, Lincoln, Nebraska, United States of America; 2 Nebraska Game and Parks Commission, Lincoln, Nebraska, United States of America; 3 Olsson Associates, Lincoln, Nebraska, United States of America; 4 U.S. Fish and Wildlife Service, Rainwater Basin Joint Venture, Grand Island, Nebraska, United States of America; 5 U.S. Geological Survey, Nebraska Cooperative Fish & Wildlife Research Unit, University of Nebraska-Lincoln, Lincoln, Nebraska, United States of America; University of California Santa Cruz, UNITED STATES

## Abstract

Understanding species-habitat relationships is vital to successful conservation, but the tools used to communicate species-habitat relationships are often poorly suited to the information needs of conservation practitioners. Here we present a novel method for translating a statistical species-habitat model, a regression analysis relating ring-necked pheasant abundance to landcover, into an interactive online tool. The Pheasant Habitat Simulator combines the analytical power of the R programming environment with the user-friendly Shiny web interface to create an online platform in which wildlife professionals can explore the effects of variation in local landcover on relative pheasant habitat suitability within spatial scales relevant to individual wildlife managers. Our tool allows users to virtually manipulate the landcover composition of a simulated space to explore how changes in landcover may affect pheasant relative habitat suitability, and guides users through the economic tradeoffs of landscape changes. We offer suggestions for development of similar interactive applications and demonstrate their potential as innovative science delivery tools for diverse professional and public audiences.

## Introduction

Conservation requires understanding relationships between species and their environments, but research concerning species-habitat relationships is often communicated through science outlets poorly suited to the information needs of conservation practitioners who implement management actions [1],[2]. Conservation practitioners are more likely to base management decisions on internal monitoring and research programs than on statistical models from the primary literature because primary literature is difficult to access and apply [3]. Journal articles are costly and time-consuming to access outside of academic institutions, especially for conservation practitioners working within state and federal natural resources agencies, and those in developing countries [4],[5]. Conservation practitioners may also perceive statistical models from the primary literature as less relevant to conservation objectives than internal agency research conducted in their own systems [6],[7]. Published species-habitat models are consequently seldom used to inform conservation actions, and practitioners rarely publish the results of management actions, limiting feedback between academic researchers and conservation practitioners [8],[9]. The implementation gap between conservation science and practice results not from a lack of interest in science by practitioners, but from a communication disconnect between researchers under institutional pressure to publish in high-impact journals and conservation practitioners striving to make the best use of limited time and money while serving the needs of diverse stakeholders [10], [11],[12]. When research is presented in a more accessible form, it is readily incorporated into management decisions [13]. Researchers also benefit from improving communication with conservation practitioners, as management agencies may be more willing to establish collaborations and provide future research opportunities to academics who present accessible results in a format readily applicable to management. The increasingly complex challenges facing natural systems demand that management actions be grounded in rigorous science, making it imperative that researchers develop effective alternatives for delivering scientific information to conservation practitioners outside the primary literature while respecting and engaging practitioners’ institutional knowledge and experience.

Statistical species-habitat models relate the spatial distribution of a species of interest to environmental variables such as geology and human infrastructure, providing spatially explicit descriptions of species-habitat relationships for ecological inference and conservation planning [10]. Species-habitat models can facilitate conservation planning by elucidating species-habitat relationships and predicting where a species is likely to be found based on known information about the environment [11],[12]. Such models can also help conservation practitioners plan for a future characterized by accelerating climate and land use change by projecting population distribution and abundance under alternative future conditions [11],[14]. Practitioners equipped with predictive information about how future environmental change may influence populations can proactively work to mitigate the potential effects of a changing landscape.

Although species-habitat models are clearly applicable to conservation, information is often presented as regression equations or static thematic maps of a large geographical area. It would be difficult for anyone to directly visualize the application of a regression equation to a landscape of interest. Likewise, static graphics too often provide little practical insight because they do not offer enough fine-scale detail that may influence local (i.e., under the supervision of an individual practitioner) management decisions [1],[15]. Furthermore, static graphics do not facilitate scenario planning using the internal research and long-term datasets maintained by many natural resources agencies. Finally, static presentations of species-habitat models do not address the economic tradeoffs of prospective management actions, a constraint experienced at every level of conservation.

To address the implementation gap between published models and management decisions, conservation organizations are increasingly interested in creating decision support tools which provide practical information from scientific studies in formats that are accessible and easy to manipulate [16],[17],[18]. As interactive web-based platforms become more widely accessible and cost-effective, researchers should enhance the utility of species-habitat models by creating interactive decision support tools that conservation practitioners can use to predict the outcomes of management actions at the spatial scale for which they are responsible [19]. Here we present a case study in which an interactive decision support tool was built at the request of a natural resources agency to address an agency-identified implementation gap between upland gamebird habitat research and management. The Pheasant Habitat Simulator is an open-source, web-based application that allows users to query and interact with a peer-reviewed species-habitat model for ring-necked pheasant (*Phasianus colchicus*) at a spatial scale relevant to on-the-ground management [20]. We present the Pheasant Habitat Simulator with its full code and data as an example framework that may be modified to accommodate a wide array of statistical models and conservation challenges.

## Methods

### Study system

Ring-necked pheasants are a culturally and economically important gamebird, naturalized and widespread across the United States. Despite the considerable scientific and management effort dedicated to North American pheasant conservation, pheasant populations continue to decline, largely due to changes in land use and agricultural practices [21],[22]. Pheasant abundance is positively correlated with landscape features such as Conservation Reserve Program (CRP) grasslands and small grain agriculture, but the strength of these associations varies with spatial scale [20],[21]. Pheasant habitat is often created through partnerships and incentives. The Conservation Reserve Program, for example, incentivizes landowners to convert marginally productive agricultural lands into grassland habitat by offering a per-acre rental rate for the land removed from production for a defined period of time, often 10 years [23]. Conservation funds are limited and conservation actions are often time-sensitive, requiring that managers be provided information about the cost-effectiveness of management actions in relation to their predicted effectiveness. The Pheasant Habitat Simulator (https://pheasant.shinyapps.io/pheasanthabitatsimulator/), here presented, translates a statistical model of pheasant-habitat relationships in Nebraska into dynamic visualizations of the predicted effectiveness and financial tradeoffs of management actions via an interactive application built on an open-source web platform.

### User interface and distribution

**Fig 1:**
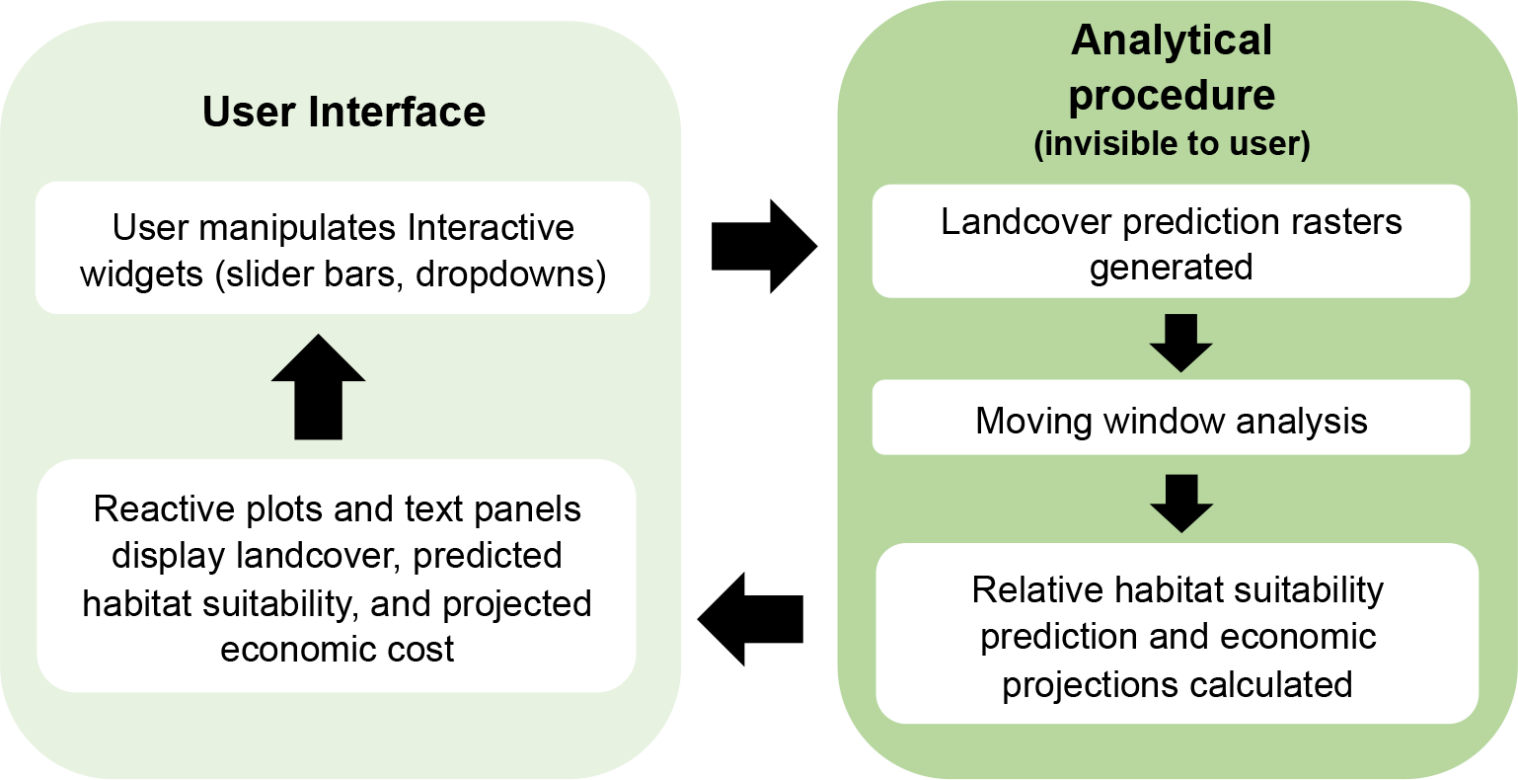
Pheasant Habitat Simulator workflow. The strength of the shiny environment is that it reactively submits the user’s land use change decisions to the analytical procedure. The analytical procedure runs in the background to render both predictions and graphical outputs without reloading the page, creating a seamless data exploration experience.

The Pheasant Habitat Simulator combines the analytical capacities of the R environment [24] and the user-friendliness of the Shiny user interface, an R package that facilitates the creation of java-based web applications (hereafter “Shiny”; [25]). The application and code presented here were developed based on open-source tools (packages: Shiny[25], dplyr [26], ggplot2 [27], raster [28], and tidyr [29]) and can thus run on most systems with sufficient memory to run basic java-based applications. We implemented a traditional analysis workflow that performs the statistical procedure underlying the model in the background of the application, invisible to the user, and updates reactively with the user’s actions. The model output is translated to a visual tool on a graphical front-end via Shiny. The strength of the Shiny application framework is its reactive programming, which links input and output data such that changes to the input results in updates to the output area without having to refresh the program, allowing users to seamlessly explore data (Fig 1). Furthermore, reactive binding between dynamic inputs and outputs enables front-end users to interact with data without requiring any knowledge of the R language. Instead, users are provided with pre-built widgets (e.g., drop-down boxes, slider bars) representing inputs that will immediately adjust linked outputs (e.g., figures, charts).

**Fig 2:**
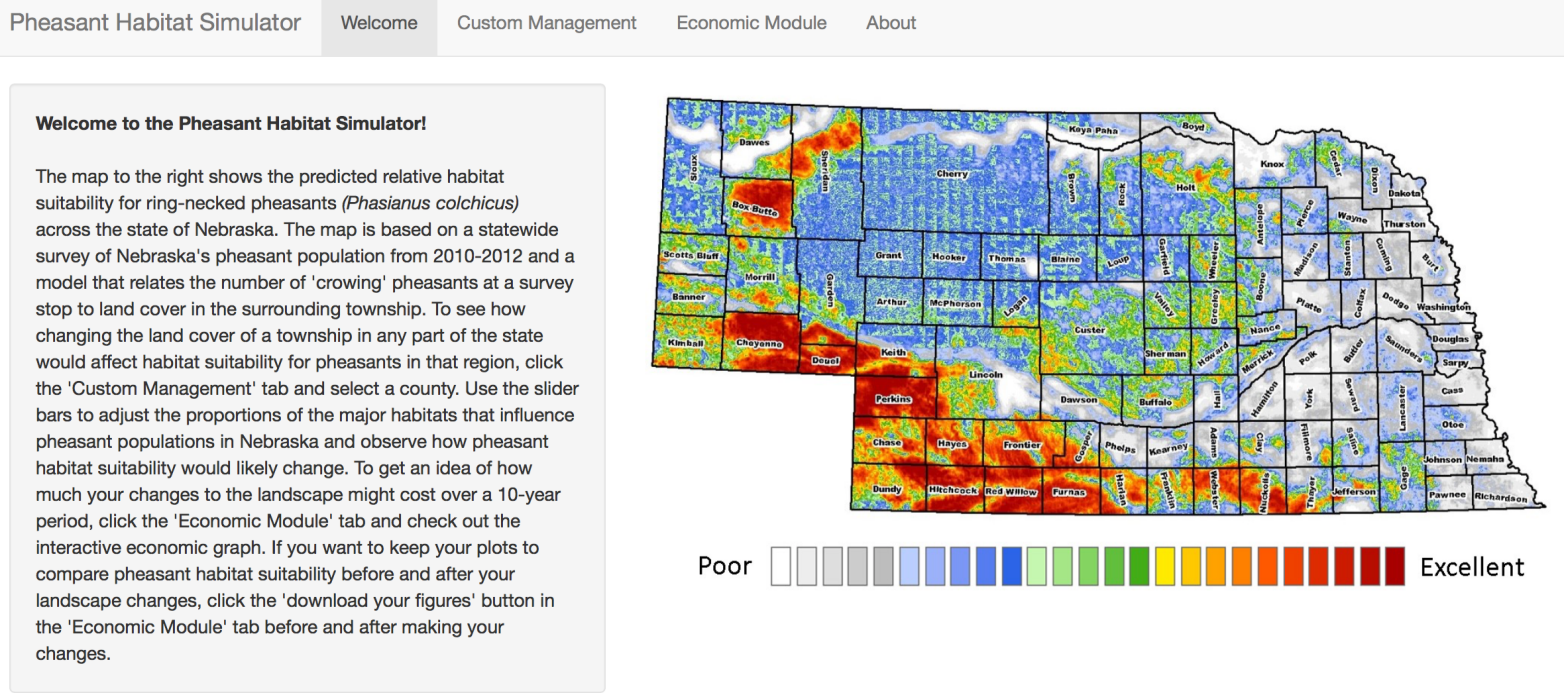
Welcome tab. The “Welcome” tab displays the current pheasant habitat suitability predictions derived from Jorgensen et al. (2014) and instructs the user on how to navigate the application.

**Fig 3:**
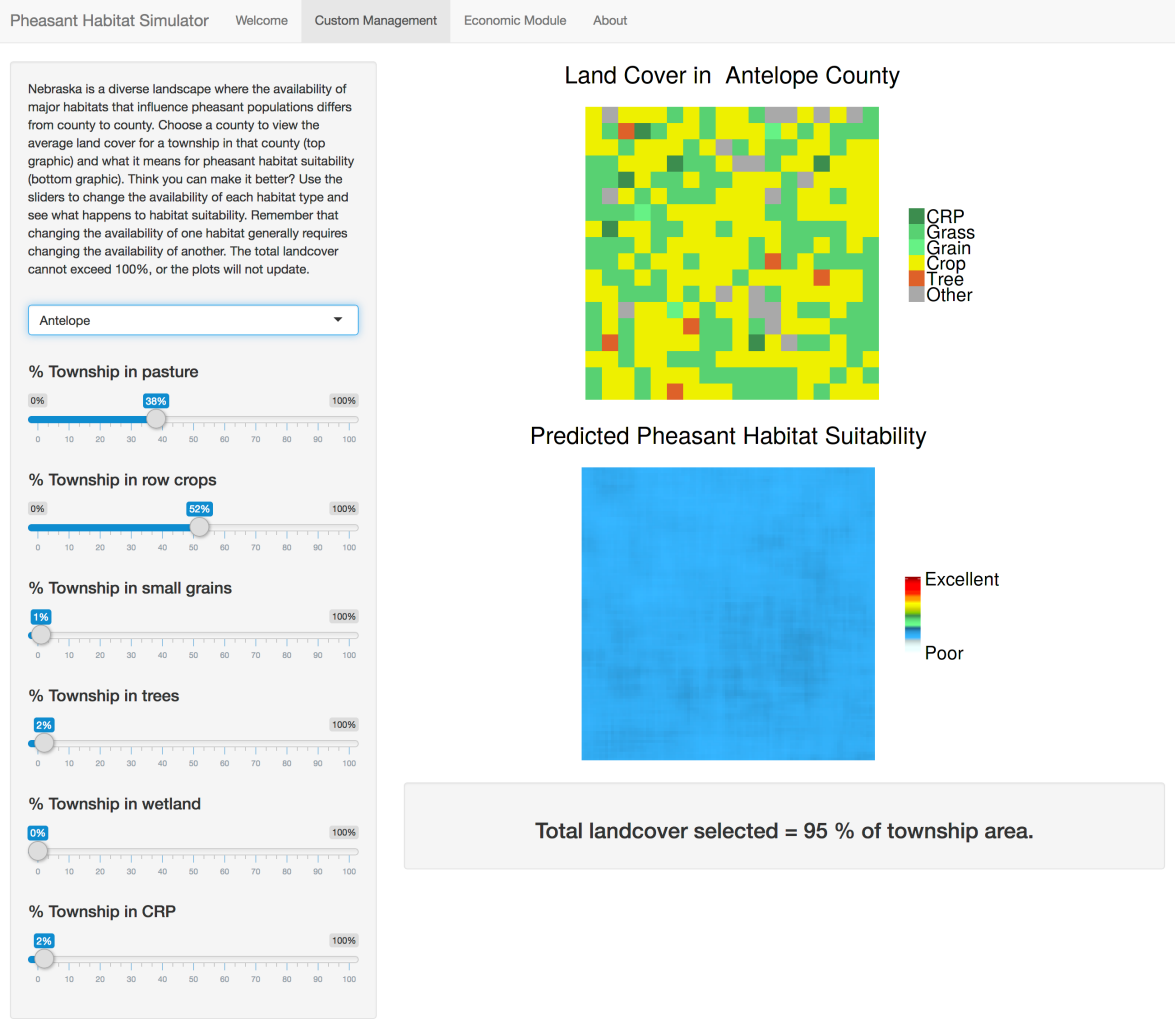
Custom management tab. The "Custom Management" tab prompts users to select a Nebraska county of management interest. The slider widgets default to reflect the average landcover of a township in that county, and the reactive plots update as the slider bars are manipulated to show landcover and predicted relative pheasant habitat suitability.

**Fig 4:**
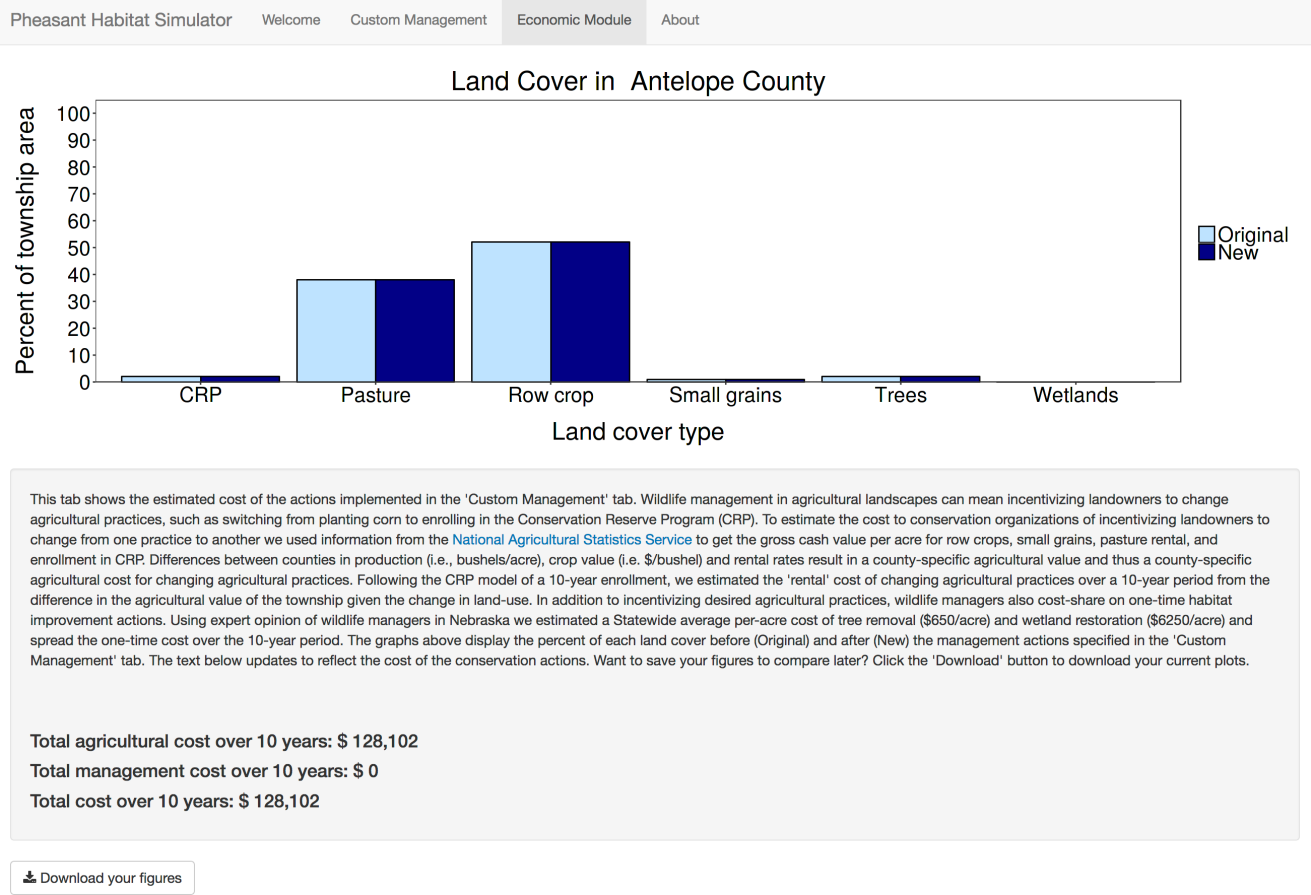
Economic Module tab. The "Economic Module" tab displays the user-specified change in landcover proportions in an updating plot. The dynamically updating text panel below the plot shows the economic implications of the user-specified landcover changes over a 10-year period, and explains how the economic analysis was conducted. Users may save their plots for viewing later using the “download your figures” button.

**Fig 5:**
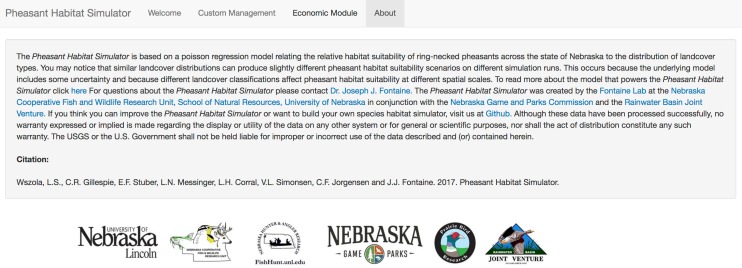
About tab. The "About" tab directs the user to the published source of the pheasant distribution model and to the websites of the developers and cooperating agencies.

The Pheasant Habitat Simulator display is a web page organized by a navigation bar with four tabs: “Welcome”, “Custom Management”, “Economic Module,” and “About” (see following sections for detail). When a user opens the application, they are directed to the “Welcome” tab, which contains a static, thematic depiction of the most recent estimated pheasant relative abundance for the state of Nebraska, and instructions on how to use the application (Fig 2). The “Custom Management” tab allows users to interact with the data entering the pheasant-habitat model by changing inputs for each predictor variable (Fig 3). We have supplied our application with two static datasets. The first dataset includes the biologically relevant landcover types: CRP grasslands, row crop, small grain, rangeland, woodland, and wetlands, that predicted pheasant abundance in the original model [20],[30] and presented here as the mean landcover proportions for each county. The mean landcover distributions appear as the default values of the dynamic slider objects once a county of interest is selected, and change when the user manipulates a slider bar. The second dataset is the county-specific economic valuations for each acre of landcover type per the National Agricultural Statistics Service. The “Economic Module” tab (Fig 4) displays the estimated monetary cost of user-defined management actions, and the “About” tab (Fig 5) includes links to the published pheasant-habitat model [20] and information about the application developers and cooperating agencies.

The Pheasant Habitat Simulator is accessible online as a webpage and a github repository (https://github.com/lsw5077/Pheasant-Habitat-Simulator). Two distribution methods provide multiple levels of engagement to suit the interests and needs of diverse users. The web-page version of the application is intended for users primarily interested in understanding pheasant-habitat relationships. For users seeking to understand the code or build their own species-habitat application, the annotated code and data are available via github. Also available at the github repository is a portable version of the app, which can be downloaded and transferred to a portable memory device or used offline on a PC (e.g., for conservation practitioners who lack a reliable internet connection or conduct meetings in the field). The portable app contains the app and the corresponding data, a copy of R portable, and a deployment skeleton that enables users to run the app simply by clicking an icon [31]. As of November 2017, the portable app has only been tested on Windows 7–10, and the deployment skeleton is only compatible with Windows. However, users running other operating systems may still access the code by navigating to the R scripts inside the portable app library. Further instructions on this procedure are included in the portable app ReadMe.

## Web interface tabs

### Custom management tab

In the “Custom Management” tab (Fig 3), users are presented with two panels: a side panel containing a drop-down menu widget that prompts users to select the Nebraska county they are interested in managing, and several slider bar widgets controlling the proportion of landcover type inputs, and a main panel containing two reactively updating plots. The widget provides the user and the model with a default landcover composition that reflects the default mean landcover composition of the townships within the chosen county and shows the user the corresponding distribution of pheasant habitat suitability categories via reactively updating plots. We chose to portray landcover composition and relative pheasant habitat suitability figures as a hypothetical “township,” (a 9324 ha block delineated by the public land survey system) to reflect the scale at which pheasants respond to many aspects of landcover [20] as well as a scale that reflects the potential for managers to act. Although the figures are visually and analytically modeled after the spatial extent of a township, the landcover figure intentionally does not represent any real place, but rather the average landscape composition of a region of interest. The model upon which the pheasant habitat simulator is based communicates the relationships between landcover composition and relative pheasant habitat suitability by producing a probability surface which is meant to be interpreted at a landscape scale [20]. The side panel directs users to move the landcover slider bars and observe how changes in the distribution of landcover categories affect the distribution of pheasant relative habitat suitability categories. Based on either county-specific default values or user-defined slider inputs, our application first generates a figure of a grid-based, habitat-classified raster reflecting the percentage of each habitat type in the “township.” For example, if the average landcover composition in the selected county contains 25% row crops and 1% CRP, then 25% of the raster pixels in the dynamically updating landcover output plot will be the “row crop” color, and 1% of the pixels will be the “CRP” color. In the background, R generates a gridded raster, the size of the “township” figure plus a 50-pixel buffer, and assigns corresponding habitat classifications to pixels proportionally according to the values entered on the reactive slider bars and randomly distributed on the grid Although the category “other” appears on the grid, because it is not a predictor of pheasant abundance, it does not enter the pheasant-habitat model, and appears in landcover figures only for visualization purposes. The application then uses a circular moving window to calculate the proportion of each habitat type within either a 10-pixel (1km) or 50-pixel (5km) radius around each focal pixel in the fine-scale background raster, as required inputs for the model upon which the application is based. Window sizes were chosen to correspond to the spatial scales of each landcover type that best predict pheasant abundance in Nebraska [20]. Because we added an additional buffer around the gridded raster, we avoid “edge-effects” associated with moving-window averages.

Because the pheasant-habitat model developed by Jorgensen et al. (2014) can be reduced to a Poisson regression equation, it is intuitive and relatively straightforward to render a pheasant habitat suitability surface according to user-specified landcover composition. Based on Jorgensen et al.’s (2014) corrected model, the Pheasant Habitat Simulator takes the proportions (un-centered and un-standardized) of rangeland (R), CRP (C), trees (T), wetland (W), and the linear, quadratic, and cubic effects of row crop (RC), and small grain (G), surrounding each pixel (p) to predict relative pheasant habitat suitability at each pixel in the fine-scale background raster:

$$\begin{array}{l} Habitat\;Suitability_{p}\\ \;=\exp(3.07+\alpha_{R}R_{p}+\alpha_{C}C_{p}+\alpha_{RC}RC_{p}+\alpha_{RC2}RC2_{p}+\alpha_{RC3}RC3_{p}+\alpha_{G}G_{p}+\alpha_{G2}G2_{p}\\ \;+\alpha_{G3}G3_{p}+\alpha_{T}T_{p}+\alpha_{W}W_{p}) \end{array}$$

This equation differs slightly from the one reported in Jorgensen et al. (2014) in that it does not include elevation, which was not determined to predict pheasant abundance. Regression coefficients were estimated accounting for imperfect detection; here, we assume constant detection probability. We use the regression equation, which was estimated from pheasant count data across the state of Nebraska, to predict the distribution of pheasant relative habitat suitability categories according to user-specified landcover composition. Predictions were based on mean parameter estimates. We do not depict statistical uncertainty because we wanted to keep the tool accessible to a broad audience, including those with limited statistical expertise. However, we have made provisions for users to investigate the role of uncertainty in model predictions to the extent that they are interested. The reactive plots are stored after each iteration, allowing users the opportunity to download individual plots to assess for themselves how similar landcover compositions may create slightly different pheasant habitat suitability surfaces. Landcover values set by the user enter the regression as variables that then predict the distribution of relative pheasant habitat suitability categories using given values for CRP, rangeland, row crops, small grains, trees and wetlands in the average township of counties throughout the state. Calculating the predictions for all pixels in the generated grid results in a predictive raster plot displayed below the landcover grid showing the distribution of relative habitat suitability categories as a probability surface that corresponds not to the landcover plot directly above it, but rather to the hidden random fine-scale raster used to predict it (Fig 3). The user thus sees a landcover composition, and a composition of habitat suitability categories which do not spatially correspond visually. When the user adjusts the landcover slider widgets, the input is reactively submitted to the regression equation workflow, which in turn updates the landcover plot and predictive pheasant habitat suitability figure. We render the plot output according to a random spatial distribution of the user’s specified landscape composition to prevent dissonance between local knowledge of landcover and the output of a landscape-scale model. The result is that the landcover and pheasant relative abundance plots are more accessible versions of bar graphs in that they display the distribution of landcover and pheasant relative abundance in a framework that is intuitive for spatially-minded users to interpret. We engineered this approach to suit the specific needs and expertise of our user base; however, the framework may be readily modified to accommodate more spatially explicit scenarios. A competent R user could quickly create rules for the distribution of landcover categories, or assign additional variables to raster layers, such as edge density or patch size if the model of interest contained such coefficients.

### Economic module tab

Wildlife managers often use financial incentives to facilitate the establishment of wildlife habitat on private lands. For example, a wildlife manager may work with a private landowner to convert cultivated land to grassland by enrolling land in CRP. Because such financial incentive programs are an important tool for wildlife management, it is essential for managers to understand the financial costs and potential trade-offs of specific land use changes designed to improve wildlife habitat suitability. The “Economic Module” tab (Fig 4) guides the user through the economic tradeoffs of the management actions specified in the “Custom Management” tab. A reactive bar graph displays the user-defined landcover types in addition to the original county default distribution of landcover types. A reactive text panel below the graph updates to reflect the user’s action on the slider widgets, calculating the monetary cost of the specified management actions over a 10-year period using county-specific economic data from the National Agricultural Statistics Service [32]. Below the reactive text panel, a download button prompts users to save their reactive plots and text elements for later study by downloading a PDF.

#### Agricultural management cost

Because CRP is one of the most important tools available to wildlife managers seeking to foster pheasant populations, the Economic Module estimates costs in 10-year increments to reflect the length of a typical CRP contract. Assuming all agricultural landcover changes result from partnerships between wildlife managers and private landowners, we estimate the incentive cost of constructing the user-defined agricultural composition as the difference between the original collective gross income to private landowners of the landscape and the collective gross income of the new, user-defined landscape, multiplied by 10 years, plus the 10-year cost of the CRP contracts. We used county-specific values for the production potential of bushels/acre and the value of crops in dollars/bushel, pasture rental rates, and enrollment rates in CRP for each county per the National Agricultural Statistics Service [32].

#### Non-agricultural management cost

In addition to incentivizing desired agricultural practices, wildlife managers also implement long-term habitat improvement actions intended to be effective for at least the duration of a CRP contract (e.g., tree removal and wetland restoration). Jorgensen et al. (2014) demonstrate that the proportion of the landscape covered in trees negatively correlates with pheasant abundance, while wetlands are positively correlated. Using expert opinion elicited from wildlife managers in Nebraska, we estimated a statewide average per-acre cost of tree removal ($650/acre) and wetland restoration ($6250/acre). The total 10-year management cost is calculated as:

$$\begin{array}{l} Total\;10-year\;management\;cost\\ \;=[\left(original\;acres\;of\;trees-user\;specified\;\;acres\;of\;trees\right)\\ \;*cost\;per\;acre\;of\;tree\;removal)]\\ \;+[\left(user\;input\;acres\;of\;wetlands-original\;acres\;of\;wetlands\right)\\ \;*cost\;per\;acre\;of\;wetland\;restoration] \end{array}$$

We assume no economic cost for increasing the proportion of trees, as planting trees for agricultural benefit is increasingly uncommon in Nebraska. We also assume no direct economic benefit to removing trees, as Nebraska does not have a largescale forestry industry. Thus, a management cost is incurred if the user decreases the proportion of trees on the simulated landscape. In contrast, we assume no cost to managers of removing wetlands, so a wetland management cost is only incurred if the user increases the proportion of wetlands within the landscape. The overall cost of creating user-defined landscape conditions is calculated by summing the total agricultural cost and the total 10-year management cost.

## Discussion

Progress in the field of conservation is hindered by a tradition of unidirectional information flow: scientific knowledge often moves, inefficiently, from scientists to conservation administrators to conservation practitioners without the opportunity for practitioners to contribute their needs and insights [1]. Web-based applications such as our Pheasant Habitat Simulator are an avenue to communicate the results of statistical species-habitat models for ecological applications while bringing scientists and conservation practitioners together as collaborators. Because web-based applications are visual and self-explanatory, there is a high return of science communicated for time invested by conservation practitioners. Web-based applications are thus readily customizable to the needs of individual user groups because they can be rapidly shared with practitioners for feedback, and then customized to the needs of the users. Such a collaborative application-development initiative can create a space where scientists and practitioners work collaboratively toward conservation goals [33].

While the case study we have presented here was created to meet a very specific need, our framework may be readily modified and improved upon to meet the needs of many professionals and researchers seeking to quickly develop management-applicable decision support tools based on statistical models. Although the model and economic calculations in our example are specific to ring-necked pheasants in Nebraska, species-habitat models are increasingly ubiquitous in the primary literature, and many rely on a data structure very similar to that used to develop the Pheasant Habitat Simulator (i.e., a regression equation describing species-habitat relationships, and suitable spatial predictor variables). Researchers or managers with some basic R coding skills, and an interest in developing a similar application can easily adjust our workflow (full code and thorough annotations are available in supporting material) to create a personalized Species Habitat Simulator specific to their region and/or species of interest. Furthermore, the dynamic user interface could be expanded to facilitate other models (e.g., occupancy, machine learning, population dynamics), for wildlife management and conservation.

Interactive decision support tools such as the Pheasant Habitat Simulator can be created and distributed through existing conservation partnerships and information channels. In many cases, administrators at conservation agencies establish objectives and conduct research, whether in house or in collaboration with an academic institution. The research team delivers findings as a research product and the administration translates findings into policies that are then implemented by conservation practitioners. We propose that interactive decision support tools such as the Pheasant Habitat Simulator represent the natural evolution of conservation research deliverables. They may be requested and produced in a similar manner to a report or presentation, with the important distinction that they may be beta tested by and customized to the needs of conservation practitioners. Furthermore, an enterprising programmer based at an agency or NGO may extract a species-habitat equation from a published manuscript and create a decision support tool for their system of interest, thus making efficient use of time and resources.

In the specific case of the Pheasant Habitat Simulator, the Nebraska Game and Parks Commission requested a decision support tool through their research partnership with the Nebraska Cooperative Fish and Wildlife Research Unit and the Rainwater Basin Joint Venture. The Pheasant Habitat Simulator was collaboratively built by graduate students and early career researchers, and then delivered to administrators at the agency, who now have the ability to widely distribute it among wildlife managers. Distribution to wildlife managers will ideally be paired with an in-person workshop or training, which can be scheduled to coincide with regional or divisional meetings when conservation practitioners convene in one location. Conservation partnerships such as the one that produced the Pheasant Habitat Simulator are widespread, with partnerships often leading to decades-long research and conservation efforts in which decision support tools are invaluable. Any organization wishing to collaborate with other conservation entities could ostensibly modify our framework to other species of conservation or management concern and host updated versions on their own website. Furthermore, because the Pheasant Habitat Simulator is an open-source tool housed on a collaborative programing website (github), we have provided the space for users to share their own improvements, large or small. The tool will thus be collaboratively improved and maintained for as long a time as it is interesting and useful. Indeed, we invite readers to provide feedback and make changes to the application on github (https://github.com/lsw5077/Pheasant-Habitat-Simulator).

Given the constraints of time and funding for development of effective science communication tools, it is essential that we streamline the process by which research results are applied to real-world problems. Researchers benefit by communicating models to practitioners effectively, as they can avoid problems caused by misinterpretation or oversimplification of ecological concepts [34]. Our example provides an approach and workflow that can be adapted to similar species-habitat relationship models. More generally, we demonstrate a framework that we hope encourages scientists to explore new avenues for communicating science to broader audiences. Web-based interactive decision-support tools provide an opportunity to reach audiences outside the scope of typical academic circles, making it more likely that the information is used in the decision making process [35]. Although until recently the development of such applications was limited to those with access to expensive software and web development expertise [36], we suggest that open-source software has made it easier for researchers to deploy model outputs. The authors of the Pheasant Habitat Simulator were able to develop and implement a decision support tool using R coding skills learned as part of regular graduate-level statistics courses. Cooperative development of interactive decision-support tools could even be integrated into a course curriculum, as students may simultaneously develop and apply statistical and programming skills to real-world conservation challenges by building decision support tools.
